# Cost and Quality Comparison of Hernia Surgery in Stationary, Day-Patient and Outpatient Care

**DOI:** 10.3390/ijerph191912410

**Published:** 2022-09-29

**Authors:** Bassey Enodien, Dominik Moser, Florian Kessler, Stephanie Taha-Mehlitz, Daniel M. Frey, Anas Taha

**Affiliations:** 1 Department of Surgery, GZO Hospital Wetzikon/Zurich, 8620 Wetzikon, Switzerland; 2 Operations Management, GZO Hospital Wetzikon/Zurich, 8620 Wetzikon, Switzerland; 3 Department of Health Care Management, Technical University of Berlin, 10623 Berlin, Germany; 4 Department of Economics and Technology, Swiss Distance University of Applied Sciences (FFHS), 8005 Zurich, Switzerland; 5 School of Medicine, University of St. Gallen, 9000 St. Gallen, Switzerland; 6 Department of Anesthesiology, GZO Hospital Wetzikon/Zurich, 8620 Wetzikon, Switzerland; 7 Department of Visceral Surgery, University Center for Gastrointestinal and Liver Diseases, St. Clara Hospital and University Hospital, 4058 Basel, Switzerland; 8 Faculty of Medicine, University of Basel, 4056 Basel, Switzerland; 9 Department of Biomedical Engineering, Faculty of Medicine, University of Basel, 4123 Allschwil, Switzerland

**Keywords:** SwissDRG, TARMED, costs, health economy, hernia, inpatient, outpatient, day clinic, cost effectiveness, quality transparency, quality, public reporting

## Abstract

Background: Medical progress is increasingly enabling more and more stationary treatment to be provided in the outpatient sector. This development should be welcomed, as healthcare costs have been rising for years. The design of efficient processes and a needs-based infrastructure enable further savings. According to international recommendations (EHS/IEHS), outpatient treatment of unilateral inguinal hernias is recommended. Method: Data from patients in GZO Hospital Wetzikon/Zurich between 2019 and 2021 for unilateral inguinal hernia repair was included in this study (*n* = 234). Any over- or under-coverage correlated with one of the three treatment groups: stationary, partially stationary and patients treated in outpatients clinic. Complications and 30-day readmissions were also monitored. Results: Final revenue for all patients is −95.36 CHF. For stationary treatments, the mean shifts down to −575.01 CHF, for partially stationary treatments the mean shifts up to −24.73 CHF, and for patients in outpatient clinic final revenue is 793.12 CHF. This result is also consistent with the operation times, which are lowest in the outpatient clinic with a mean of 36 min, significantly longer in the partially stationary setting with 58 min, and longest in the stationary setting with 76 min. The same applies to the anesthesia times and the relevant care times by the nurses as the most important cost factors in addition to the supply and allocation costs. Conclusions: We show that cost-effective elective unilateral inguinal hernia care in the outpatient clinic with profit (mean 793.12 CHF) is possible. Stationary unilateral hernia care (mean −575.01 CHF) is loss-making. Crucial factors for cost efficiency are optimized processes in the operating room (anesthesia, surgical technique and quality, operating time), as well as optimized care processes with minimal preoperative services and care times for the patient. However, at the same time, these optimizations pose a challenge to surgical and anesthesiology training and structures with high levels of preoperative and Postoperative services and pay-as-you-go costs. The complication rate is 0.91% lower than in a comparable study. The readmission within 30 days post-operation results with a positive deviation of −3.53% (stationary) and with a negative deviation of +2.29% (outpatient clinic) compared to a comparative study.

## 1. Introduction

Since 2012, stationary, acute-somatic hospital services in Switzerland have been reimbursed employing per-case flat rates, known as Diagnosis Related Groups (SwissDRG) [1]. The DRG is assigned by a grouper. This links patient data with information on the hospital stay, diagnoses, procedures, and any special medications. This results in a case severity, which is multiplied by the base rate. The base rate is negotiated by the insurance companies with the hospitals or in case of disagreement, set by the health authorities. Stationary hospital services are paid up to a maximum of 45% by the insurance companies and up to a minimum of 55% by the Swiss cantons (public government) [2]. In the outpatient sector, hospital services are billed entirely by the insurance companies based on the TARMED tariff system. The patient pays a small share of the costs in each case (deductible, franchise). The different financing leads to misaligned incentives, which will not be discussed further here.

Medical progress is increasingly enabling more and more stationary treatment to be provided in the outpatient sector. Regarding total cost accounting, this development should be welcomed, as healthcare costs have been rising for years despite this trend [3]. The outpatient, elective area allows for accurate planning of all resources. Expensive emergency reserve capacities are eliminated. Likewise, a more specific deployment of personnel is possible; unnecessary over qualification is avoided. The design of efficient processes and a needs-based infrastructure enable further savings in the outpatient sector.

Inguinal hernia care in particular is a very commonly performed operation in general surgery clinics and specialized centers. The risk of suffering from symptoms of inguinal hernia in the course of life is 27–43% in men and 3–6% in women [4,5]. Femoral hernias are less common defects and are responsible for 3% of all inguinal hernias. Femoral hernias occur significantly more often in women at a rate of 10:1.

Inguinal hernias are classified according to the European Hernia Society as medial, lateral and femoral (M/L/F), according to size as 1 (max. 1 finger), 2 (1–2 fingers) and 3 (3 fingers or more), as well as primary hernias (P) and recurrences (R) [6,7].

According to the recommendations of the European Hernia Society (EHS) and the International Endohernia Society (IEHS), outpatient management of unilateral inguinal hernias is recommended [5,7,8]. A standardized technique is recommended for management, with open mesh reinforcement as per Liechtenstein, as well as both endoscopic techniques with extraperitoneal preperitoneal and transabdominal preperitoneal mesh reinforcement considered equivalent and standard treatments. Additionally, the definition and classification according to EHS favor the standardization of the treatment and thus both process optimization and quality improvement.

In our previously published study “Analysis of Factors Relevant to Revenue Enhancement in Hernia Interventions (SwissDRG G09)”, we analyzed the factors that are relevant to revenue enhancement in hernia surgery in the stationary setting. Our analysis showed that there is an average loss of −623.84 CHF per case in the stationary setting for this treatment [6]. The relevant factors that could be influenced (not demographic) to increase yield were surgical teaching during surgery, surgical operating time, total anesthesia time, number of surgeons present (resources), and surgical technique with the Lichtenstein open technique shown to be more cost effective.

For the present study, we focused on a comparison of care options in an stationary and outpatient setting in a day clinic and in a process-optimized outpatient clinic according to Taiichi Ohno’s lean management philosophy [9,10,11]. The goal of this optimization based on the lean management philosophy is to control quality through process optimization and to simultaneously improve financial performance, such as through the targeted use of all resources and standardization. The significant findings described above for increasing earnings have already been taken into account in the treatment of patients in the present analysis [8,12,13].

The Swiss Federal Health Insurance Act (KVG) stipulates that services must always be effective, appropriate and economical [14]. The law thus expects quality to be taken into account in the further development of treatment processes. According to Donabedian, this includes not only structural quality (e.g., sufficient availability of specialist staff) but also process and outcome quality [15]. However, the evaluation of treatment optimization in terms of effectiveness, appropriateness and cost-effectiveness between the inter-hospital inpatient and outpatient sector is currently not possible in Switzerland [16]. For example, patient and treatment data from different health care providers in the outpatient and stationary settings cannot be linked to enable treatment evaluations. Due to the information asymmetry between patients and providers, quality data, the public and critical discussion about them, and benchmarks make an important contribution to quality transparency [17]. In the future, this will allow the increased inclusion of outcome quality for health care planning to enable the balanced consideration of all three factors (effective, appropriate, economic) and to reduce quality variation [18]. Therefore, in the present study, we would like to contribute to quality transparency by including the following quality indicators: Unplanned readmissions within 30 days of discharge and treatments planned for outpatients who ultimately unexpectedly required stationary care anyway. Junaid et al. [19] described complication rates of 10% with urinary retention, 6.6% with seroma formation, and 3.3% each with wound infection and persistent pain. Friedlander et al. [20] observed a 2.9% readmission rate within 30 days in 2131 elective index cases (stationary: 5.8%, outpatient clinic: 2.5%). Due to the small number of cases in this study, the types of complications are not broken down (chronic postoperative pain, wound infections, urinary and sexual dysfunction, hematomas, seromas, etc.) [5].

In general, outpatient inguinal hernia operations reimbursed according to the TARMED tariff generate 1.9 to 3.2 times less revenue in this system than stationary treatments reimbursed at a flat rate according to the SwissDRG system [21]. A 2016 study by PricewaterhouseCoopers (PWC) estimates the potential savings of this measure in the Swiss healthcare system at 1 billion CHF per year if implemented consistently in all areas. According to the Federal Statistical Office (FSO), total healthcare spending in Switzerland amounted to 82.5 billion CHF in 2019, representing 11.3% of Gross Domestic Product (GDP) [22].

Therefore, an increase in the proportion of outpatient surgeries at the expense of stationary treatments is seen as a goal to reduce healthcare spending by politicians and health insurance providers. Since the introduction of Diagnosis-Related Groups in Switzerland (SwissDRG), hospitals have been under system-related optimization pressure. The increasing shift from stationary to outpatient treatment poses an additional challenge [6,23]. The COVID 19 pandemic has also led to unexpected revenue shortfalls for healthcare institutions worldwide [24]. In Switzerland, the shortfall in revenue is estimated to be between 0.9 and 1.1 billion CHF [21].

The pressure to optimize is forcing hospitals to continuously improve their own processes and structures. The present study was conducted at GZO Hospital Wetzikon/Zürich, a center hospital in Switzerland with about 10,000 stationary cases. In 2019, the hospital started building an outpatient clinic modeled after a Danish university hospital. Processes were fundamentally redesigned and implemented, always aiming for the highest effectiveness with the highest possible efficiency. Our hypothesis: With an outpatient clinic optimized according to the lean management philosophy, elective inguinal hernia surgery, among other procedures, can be performed more economically than in the existing structures. Previously, stationary treatment was possible as well as outpatient treatment via the day clinic. Both options, however, have a wide range of services and corresponding reserve capacities, such as blood sampling at the day clinic for other clinics. It is to be expected that these same reserve capacitieswill increase the costs of individual cases or that cost allocations will be reported to the disadvantage of elective surgeries. The outpatient clinic, on the other hand, is entirely focused on early planned operations and all processes have been interdisciplinary optimized for quality stability and efficiency.

## 2. Method

### 2.1. Data Collection

Data from all the patients admitted to GZO Hospital Wetzikon/Zürich between January 2019 and December 2021 for unilateral inguinal hernia repair were included in this study (*n* = 234; stationary = 88, partially stationary = 107, outpatient clinic = 39). We were able to obtaine data from the controlling department’s internal data processing system and correlated these with the definitive contribution margin (CM) of individual procedures. The CM value indicates a possible over or under-coverage relating to case-specific costs and is referred to hereinafter as final revenue. To achieve the base price of a DRG case-based lump sum we multiplied the respective evaluation ratio by the base case rate. Any over- or under-coverage correlated with one of the three treatment groups was recorded (stationary treated patients, partially stationary treated patients, outpatients clinic). Quality information was added manually. The GZO hospital Wetzikon/Zurich is a private hospital, owned by the local communities, with a public service contract. Final revenue is shown in CHF. The current exchange rate to the EUR is 1:1.

### 2.2. Statistical Analysis

A linear regression model was created in which the dependent variable *Final Revenue* (income after deducting all costs in CHF) was explained with the following twelve predictor variables: *age* (in years), *doctors costs* (in CHF), *material costs* (in CHF), *surgery time* (in min), *anesthesia time* (in min), *stationary care time* (in min), *outpatient clinic care time* (in min), *recovery room care time* (in min), *stay type* (with three categories: stationary/partially stationary/Outpatient clinic), *surgery type* (open surgery/laparoscopic surgery), *incarcerated hernia* (yes/no), and *insurance type* (general/private). To compare the three types of hospital stay, interaction terms of the first and second order with the variable *stay type* were added to the model. Another variable in the model is gender. For ethical reasons, we have chosen not to report gender differences in costs and benefits.

The analyzes were carried out in the R computing environment version 4.1.2 [25]. Interaction diagrams and simple slopes [26] were computed with the R package interactions [27].

## 3. Results

### 3.1. Descriptive Statistics

Descriptive statistics for the whole sample and for the subgroups of the stay types are shown in Table 1.

**Figure 1:**
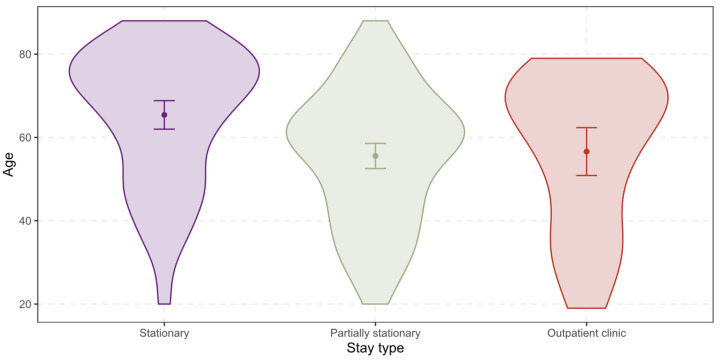
Differences between the stay types regarding the age of the patients.

The *age* ranges from 19 years to 88 years with a mean of 59.4 years and a standard deviation of 17.1 years for all patients (see Figure 1 below). For stationary treated patients, the mean shifts up to 65.4 years (SD = 16.4 years), while for partially stationary treated patients the mean shifts down to 55.6 years (SD = 16.0 years), and for patients in outpatient clinic to 56.6 years (SD = 18.3 years).

**Figure 2:**
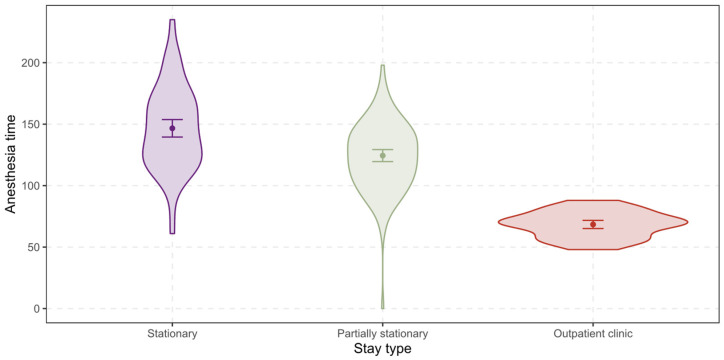
Differences between the stay types regarding the anesthesia time.

The *anesthesia time* ranges from 0 min to 235 min with a mean of 123 min and a standard deviation of 39 min for all patients (see Figure 2 below). For stationary treated patients, the mean shifts up to 147 min (SD = 34 min), while for partially stationary treated patients the mean remains stable at 124 min (SD = 26 min), but for patients in outpatient clinic it shifts down to 68 min (SD = 11 min).

**Figure 3:**
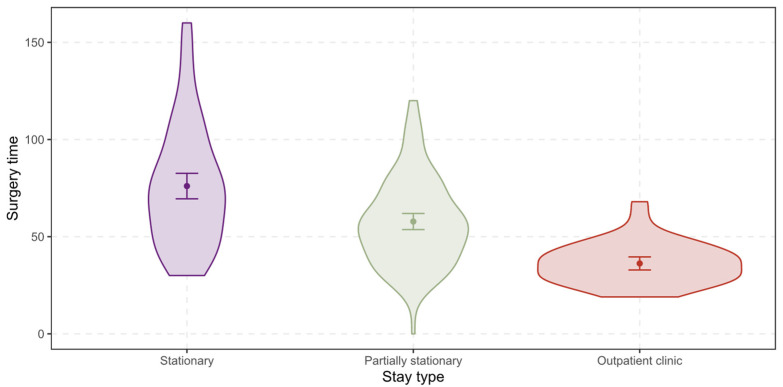
Differences between the stay types regarding the surgery time.

The *surgery time* ranges from 0 min to 160 min with a mean of 61 min and a standard deviation of 28 min for all patients (see Figure 3 below). For stationary treated patients, the mean shifts up to 76 min (SD = 31 min), while for partially stationary treated patients the mean shifts down to 58 min (SD = 22 min), and for patients in outpatient clinic to 36 min (SD = 11 min).

The *doctors costs* ranges from 72.47 CHF to 6280.61 CHF with a mean of 855.47 CHF and a standard deviation of 822.12 CHF for all patients. For stationary treated patients, the mean shifts down to 686.32 CHF (SD = 372.17 CHF), for partially stationary treated patients to 654.96 CHF (SD = 201.98 CHF), and for patients in outpatient clinic to 687.07 CHF (SD = 138.81 CHF).

The *material costs* ranges from 246.63 CHF to 3089.02 CHF with a mean of 672.11 CHF. For stationary treated patients, the mean shifts up to 1595.77 CHF (SD = 926.86 CHF), for partially stationary treated patients down to 409.14 CHF (SD = 170.52 CHF), and for patients in outpatient clinic to 411.99 CHF (SD = 253.72 CHF).

**Figure 4:**
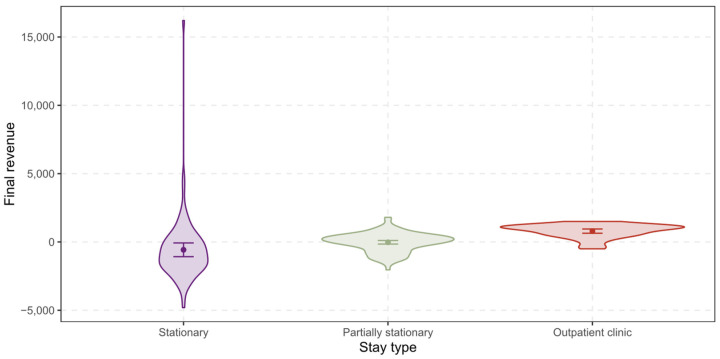
Differences between the stay types regarding the final revenue.

The *final revenue* ranges from −4807.04 CHF to 16,207.03 CHF with a mean of −95.36 CHF and a standard deviation of 1624.42 CHF for all patients (see Figure 4 below). For stationary treated patients, the mean shifts down to −575.01 CHF (SD = 2405.49 CHF), for partially stationary treated patients the mean shifts up to −24.73 CHF (SD = 690.76 CHF), and for patients in outpatient clinic to 793.12 CHF (SD = 493.90 CHF).

As shown in Table 2, the statistics on care times depend on the extent to which the various types of care were used. Not all patients spent time in the recovery room and after that, further care was taken over by the department or the outpatient clinic or both. Therefore, the statistics on the care times are based on different number of patients.

**Figure 5:**
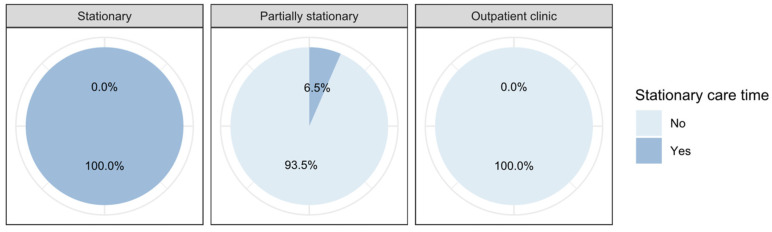
Differences between the stay types regarding the care in the department.

The *stationary care time* ranges from 0 min to 929 min with a mean of 165 min and a standard deviation of 224 min for all patients (see Figure 5 below). For stationary treated patients, the mean shifts up to 425 min (SD = 148 min), while for partially stationary treated patients the mean shifts up to 186 min (SD = 60 min). Patients in the outpatient clinic are not nursed in the department.

**Figure 6:**
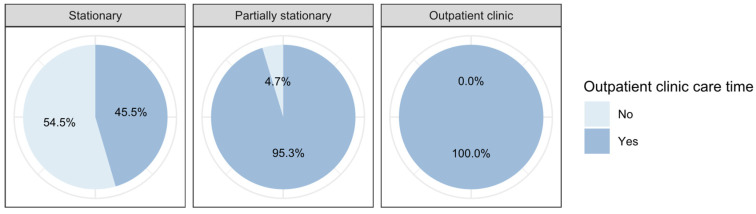
Differences between the stay types regarding the care in the day clinic.

The *outpatient clinic care time* ranges from 0 min to 300 min with a mean of 45 min and a standard deviation of 39 min for all patients (see Figure 6 below). For stationary treated patients, the mean shifts slightly up to 47 min (SD = 34 min), while for partially stationary treated patients the mean shifts up to 61 min (SD = 36 min), and for patients in outpatient clinic to 60 min (SD = 31 min).

**Figure 7:**
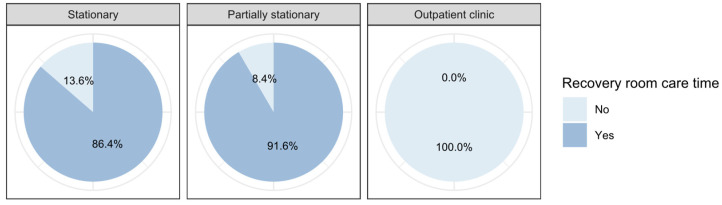
Differences between the stay types regarding the care in the recovery room.

The *recovery room care time* ranges from 0 min to 230 min with a mean of 61 min and a standard deviation of 47 min for all patients (see Figure 7 below). For stationary treated patients, the mean shifts up to 99 min (SD = 36 min), while for partially stationary treated patients the mean shifts up to 68 min (SD = 27 min). The recovery room is not used for patients in the outpatient clinic.

#### Quality Indicators

Of the 88 stationary treatments, 16 were primary emergencies (18.18%), and another 8 patients were admitted to stationary care on the day of surgery after initially planned outpatient treatment (9.09%). The reasons were postoperative pain, intraoperative complications, or complications affecting the cardiovascular system. There were no readmissions after discharge among stationary treated patients up to 30 days postoperatively (0.0%). However, there were 2 emergency outpatient consultations after stationary stay (2.27%). Among the total 146 outpatients (partially stationary, Outpatient clinic), 7 were checked unplanned in the emergency department postoperatively (4.79%), but there was no stationary readmission within 30 days (0.0%).

### 3.2. Moderated Multiple Linear Model

#### 3.2.1. Model Assessment

The set of 12 predictors used in the linear model was carefully chosen from a set of 26 variables. As the tolerance values presented in Table 3 show, most predictors hardly share their variance with other predictors, thereby providing unique variance for the model. Due to this careful selection and the relatively large sample size of 234 observation cases, a moderated multiple linear model containing interaction terms of the first and second order with the categorical variable *stay type* could be developed that almost completely explains the variance of the dependent variable *Final Revenue*.

The final model (see Table 4) achieved a fit of $r^{2}$ = 0.9782 (corrected 0.9605), which can be described as excellent. The requirements for a valid regression model with regard to homoscedasticity and normal distribution of the residuals are met (score test for non-constant error variance: p = 0.618, Shapiro–Wilk normality test: p = 0.261). A power analysis with G*Power 3.1.9.7 [28] reveals that the model has a power of 0.94 to detect large effects from $f^{2}$ = 0.35 and a power of nearly 0.80 to detect medium effects from $f^{2}$ = 0.25. Significant results from Table 4 are described in the following sections.

#### 3.2.2. Surgery Type

**Figure 8:**
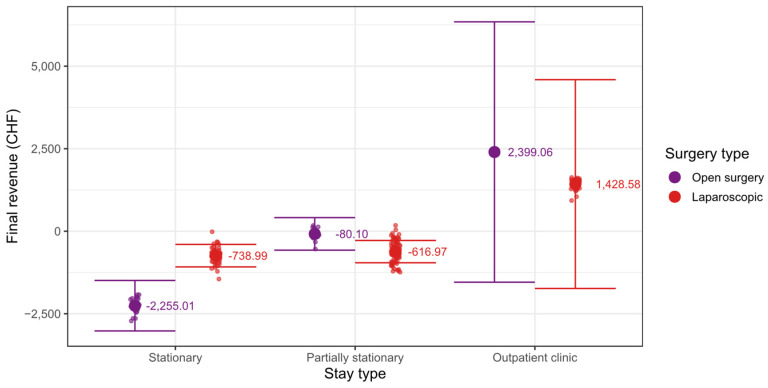
Surgery type mean differences in the stay type groups.

The differences between the type of surgery depend on the type of hospital stay as shown in Figure 8.

In the case of stationary treatment, there is a significantly lower final revenue of −2255.01 CHF for the open surgery compared to the final revenue of −738.99 CHF for the laparoscopic surgery (b = 1516.01; β = 0.93; p < 0001). In the case of partially stationary treatment, the final revenue of −80.10 CHF for the open surgery is significantly higher than the final revenue of −616.97 CHF for the laparoscopic surgery (b = −536.87; β = −0.33; p = 0.035). For treatment in the outpatient clinic, there is no significant difference between the final revenue of 2399.06 CHF for the open surgery and the final revenue of 1428.58 CHF for the laparoscopic surgery (b = −970.48; β = −0.60; p = 0.209). However, the result is non-significant due to the fact that there is only one case in the open surgery group in the outpatient clinic (see Table 5).

The beta weight of 0.60 indicates a medium-sized effect that would have become significant with more open surgery cases from the outpatient clinic.

The interaction between the type of surgery and the type of stay can be described as follows. The difference in final revenue between open and laparoscopic surgery is significantly larger in the stationary treatment group than in partially stationary treatment group (b = 2052.88; β = 1.26; p = 0.001). The difference is also significantly larger in the stationary treatment group than in the outpatient treatment group (b = 2486.49; β = 1.53; p = 0.012).

#### 3.2.3. Surgery Time

**Figure 9:**
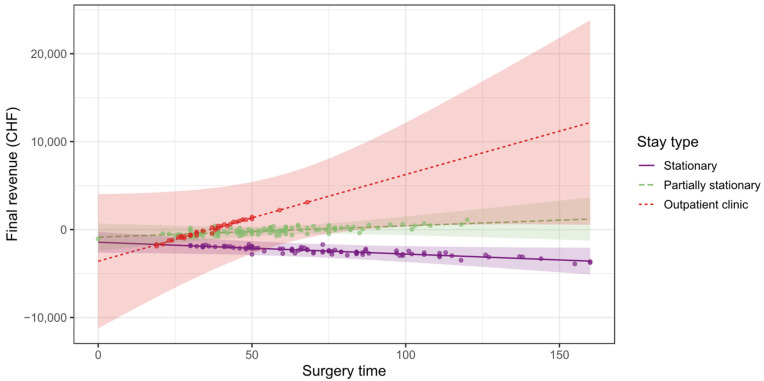
Dependence of final revenue on surgery time.

As shown in Figure 9, the impact of surgery time on the final revenue depends significantly on the type of hospital stay. In the case of stationary treatment, the final revenue does not significantly decrease by b = 4.04 CHF for every minute of surgery time (β = −0.07; p = 0.587). In the case of partially stationary treatment, the final revenue increases almost significantly by b = 22.32 CHF with every minute of surgery time (β = 0.39; p = 0.073). Treatment in the outpatient clinic has the greatest impact on the final revenue: the final revenue increases significantly by b = 108.01 CHF per minute of operation time (β = 1.89; p = 0.047).

#### 3.2.4. Impact of Surgery Time Depending on the Age of the Patients

**Figure 10:**
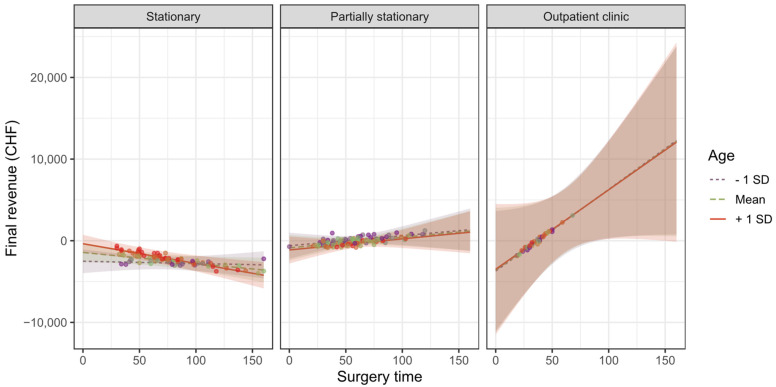
Dependence of final revenue on surgery time and age.

As depicted in Figure 10, the dependence of the final revenue on the surgery time is moderated by the patient’s age, but only for stationary treatment. In this case, the amount by which the reduction in final revenue occurs increases with the age of the patient, so that from the age of 77 a significant reduction of b = −14.69 CHF per minute of surgery time can be expected (β = −0.26; p = 0.051).

#### 3.2.5. Outpatient Clinic Care Time

**Figure 11:**
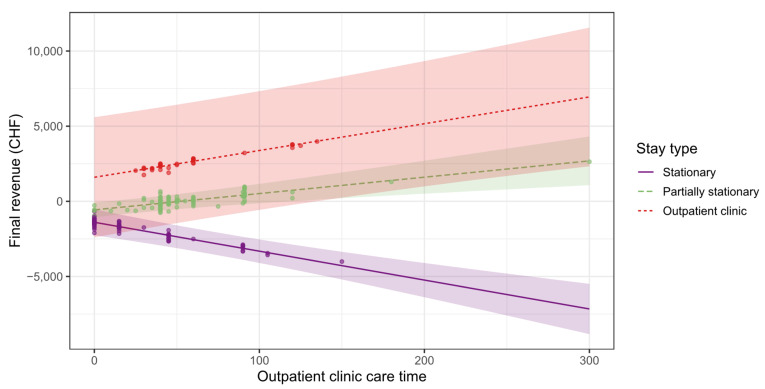
Dependence of final revenue on outpatient clinic care time.

There are differences in the outpatient clinic care time depending on the type of stay, as shown in Figure 11 and Table 5. Around 45% of the stationary treated patients are finally cared for in the outpatient clinic (see Table 2), which significantly reduces the final revenue by b = −19.76 CHF per minute of care in the outpatient clinic (β = −0.48; p < 0.001). On the other hand, outpatient clinic care time of partially stationary treated patients (b = 10.34; β = 0.25; p < 0.001) and outpatients (b = 17.29; β = 0.42; p < 0.001) leads to a significant increase in the final revenue.

## 4. Discussion

Despite various measures implemented by the government, health care costs in Switzerland continue to rise [21]. This is not a regional but a global problem. Medical progress, unhealthy lifestyles and demographic change are leading to rising healthcare expenditure in many countries [29]. The WHO 2020 Report, which analyzed health spending in 190 countries from 2000 to 2018, showed continuously increasing health spending globally, reaching 8.3 trillion USD or 10% of global GDP in 2018 [29]. Therefore, as described in the introduction, there is great pressure on healthcare providers to reduce costs and increase efficiency while improving quality. A study published in 2019 by Friedländer et al. in the annals of surgery [20], which compared the costs of outpatient and stationary care for hernias and other conditions, showed significantly lower costs for outpatient hernia care. In our comparison between stationary, partially stationary and hernia care in the specialized outpatient clinic, the mean final revenue for all patients was −95.36 CHF. For stationary treated patients, the mean shifts down to −575.01 CHF. This corresponds to the results of Raakow et al. in 2019 in “Journal der Chirurgie” in their publication “Elektive Versorgung von Leistenhernien in der universitären Chirurgie-eine ökonomische Herausforderung” (Elective care of inguinal hernias in university surgery—an economic challenge), which showed a deficit of 651 EUR per case in elective hernia care. Significant factors were defined as postoperative complications, operating time, anesthesia time, and nursing, maintenance, and apportionment costs [30].

The difference to the current results can be explained by the fact that in the previous analysis only stationary treatments were analyzed and that the knowledge gained on revenue enhancement has already been implemented to a large extent in the treatments now under consideration. For partially stationary treated patients the mean shifts up to −24.73 CHF. This distinct cost advantage, with increasing cost pressure and the development towards outpatient care was already predicted by the world-famous hernia surgeon Prof. Dr. V. Schumpelick in his 2004 article “Surgery of inguinal hernia as ambulatory and brief stationary surgery” [8]. A unique result that we could present in our analysis is that for patients in outpatient clinic the net revenue is 793.12 CHF. For the first time, this demonstrates that cost-efficient elective inguinal hernia care is possible in an outpatient clinic optimized according to the lean management philosophy, contrary to what was described by Raakow et al. [30]. A further study by McCormack et al. in 2010 described open inguinal hernia surgery according to Lichtenstein as more cost-effective compared with endoscopic techniques [31].

This result is also consistent with the operation times, which are lowest in the outpatient clinic with a mean of 36 min (SD = 11 min), already significantly longer in the partially stationary setting at 58 min (SD = 22 min), and longest in the stationary setting at 76 min (SD = 31 min). Regarding these results, it must be said that in the outpatient clinic, teaching is limited to the specialized training of an already certified specialist in the field of hernias. For reasons of efficiency, operations accompanied by beginners are not performed in the setting of the outpatient clinic. On the one hand, operations can be performed in a shorter time without any loss of quality, and on the other hand, changes in the surgical team are deliberately avoided. In this setting, the same team operates all day long, which results in a cadence of surgeries that beginners are not yet capable of handling. This loss of operations suitable for teaching due to measures to increase yield and quality is a major and unresolved problem in surgical education today [32,33]. Same applies for anesthesiology training. In terms of Anesthesia times these are also lowest in the outpatient clinic with 68 min (SD = 11 min), followed by the partially stationary setting with 124 min (SD = 26 min) and the stationary setting with 147 min (SD = 34 min). It must be mentioned that the operation time is a subset of the anesthesia time. However, even when subtracting the surgery time, the same difference remains. With regard to the anesthesia time, it must be added that in the outpatient clinic special anesthesia procedures are used to ensure short anesthesia times. Especially the skill-grade mix of anesthesia differs from the day program at the GZO Hospital in Wetzikon/Zurich in the Outpatient Center (OC).

In the regular day program, an average of four operating rooms (OR) are operated. Two senior physicians are available for running the center. For each OR, there is also a certified expert in anesthesia care together with an assistant physician. They alternate in the day program with the anesthesia management of the patients. The senior physician is present during induction and discharge and can be called in intraoperatively by the anesthesiologist at any time.

By comparison, the OC-OR track is supervised by a senior physician along with a certified expert in anesthesia care. Therefore, the presence of the assistant physician is usually omitted. The senior physician, however, is present at all times.

The use of specialist physicians and anesthesia care nurses with long-term professional experience means that the anesthesia can be discharged punctually at the end of the intervention. The number of anesthesia overruns is reduced. Due to the “point landings”, the OR is quickly available again for the next intervention. As a result, there is no need to exit the room with intubated patients compared to the day program.

In order to make the anesthesia team available again as quickly as possible, patients are picked up by the recovery room team directly at the OR while the next patients are brought to the OR, whereas in the day program the anesthesia team is responsible for bringing the patients to the recovery room. After a short preparation of the workstation, the next anesthesia induction can be started promptly in the recovery room.

In order to further optimize the transition times in the recovery room, patients are brought to the operating room with a peripheral venous catheter (PVK) already in place. The recovery room team places this in advance. A disposable blood pressure cuff is also installed for monitoring.

The age of the patients in the outpatient clinic with 56.6 years (SD = 18.3 years) and in the partially stationary setting with 55.6 years (SD = 16.0 years) is not relevantly different. While the patients in the stationary setting are significantly older with 65.4 years (SD = 16.4 years). This indicates that patients in the stationary setting are more likely to show comorbidities due to their age, which in turn qualifies them for the stationary stay and also affects the anesthesia time based on the ASA classification (American Society of Anesthesiologists) [34]. However, in the stationary SwissDRG system, increased age and additional comorbidity are compensated with increased final charges, which have already been considered in the present study [6]. In general, outpatient treatment is feasible for any patient regardless of gender or age. However, the following patient-specific factors make inpatient treatment necessary. Expected intensive care follow-up. Nontemporarily substitutable blood disorders, oral anticoagulation, coagulopathy relevant to surgery and use of drainage tubes. Comorbidities such as significantly pathologic pulmonary parameters, known severe obstructive sleep apnea disease, angina pectoris grade III or IV (CCS), heart failure NYHA III or higher. Malignant hypertermia in self history or family history, obesity WHO grade III or other severe unstable diseases. Social factors due to which immediate medical care of the patient would not be possible such as, Lack of communication facility, no transportation facility to or poor accessibility of medical care Lack of patient’s ability to understand, lack of care facility by person in the first 24 h after surgery. These factors correspond to the rules which apply to the inpatient treatment of a patient with elective inguinal hernia care in our hospital. Emergencies are always treated as inpatients. These factors are often but not always associated with older age.

Concerning physician costs, it is notable that these are similarly high in all areas: 686.32 CHF (SD = 372.17 CHF) for stationary treated patients, 654.96 CHF (SD = 201.98 CHF) for partially stationary treated patients and 687.07 CHF (SD = 138.81 CHF) for patients in outpatient clinic. However, there are significant differences in material costs with 1595.77 CHF (SD = 926.86 CHF) for stationary patients, 409.14 CHF (SD = 170.52 CHF) for partially stationary treated patients and 411.99 CHF (SD = 253.72 CHF) for patients in outpatient clinic. These increased costs for material consumption can be explained by the increased expenditure during a stationary stay, which is why the stationary stay is reimbursed 1.9–3.2 more highly compared to the outpatient stay [21]. Nonetheless, our analysis reveals that stationary treatment involves at least 3.9 times higher material costs compared with outpatient treatment. In the care times provided by nurses in the different departments, further differences emerge, which may explain the increased costs in stationary and the lower costs in the partially stationary and outpatient setting, as well as the increased final revenue in the outpatient clinic. The mean recovery room time for stationary patients was 99 min (SD = 36 min), while for partially stationary patients the mean shifts down to 68 min (SD = 27 min). The recovery room is not used for patients in the outpatient clinic. Stationary patients were treated by nurses in the outpatient clinic for a mean of 425 min (SD = 148 min), while for partially stationary treated patients the mean shifts down to 186 min (SD = 60 min). Patients in the outpatient clinic are not nursed in this department at all. In the outpatient clinic mean care time for stationary treated patients is 47 min (SD = 34 min), while for partially stationary treated patients the mean shifts up to 61 min (SD = 36 min), and for patients in outpatient clinic to 60 min (SD = 31 min).

In addition to descriptive statistics, we developed a moderated multiple linear model, which includes interaction terms of the first and second order with the categorical variable stay type to explain the variance of the dependent variable final revenue. Regarding the surgical technique, it was shown that the final revenue in the stationary setting is significantly higher with the endoscopic technique than with the open technique according to Lichtenstein (*p* < 0.001). In the outpatient setting, however, the final revenue is significantly higher for the open surgical technique according to Lichtenstein (*p* = 0.035). A study from the USA published in 2008 by Jacobs et al. showed higher revenues for endoscopic care due to increased final revenue for this procedure in their system opposed to our results [13]. In the outpatient setting, final revenue is also higher for the open technique but not significantly (*p* = 0.209). One explanation for the lower revenue with the open technique according to Lichtenstein in the stationary setting could be that these surgeries are usually used as teaching surgeries, where lower revenue due to increased surgery time has been demonstrated [6]. An explanation for the increased yield in the outpatient setting could be the remuneration in the Swiss outpatient TARMED tariff, where the remuneration for the open technique is higher and that the outpatient surgeries are usually not teaching surgeries. Considering the operating time, the revenue decreases as expected but not significantly with each operating minute (*p* = 0.587). Contrary to expectations, revenue in the outpatient setting (*p* = 0.073) increases significantly with each OP-minute (*p* = 0.047). This could be due to the physician costs included in the cost accounting system “REKOLE^®^”, where internally different multipliers are used for the physicians depending on their level. The chief of surgery with a correspondingly higher salary receives a multiplicator of 2.5, and a chief of service 1.5, while attending surgeons receive a cost weight of 1. This can result in a higher reported revenue for an attending surgeon with a cost weight of 1 than for a chief of surgery with a cost weight of 2.5, despite a longer operation time. The higher costs for shorter operations can therefore indicate faster but more expensive surgeons (chief of surgery, chief of service). A significant relationship between the influence of variable surgery time on final revenue and the demographic factor of age could be demonstrated for stationary treated patients (*p* = 0.051), but no significant influence could be found for outpatient treatments. 45% of the stationary treated patients were additionally treated in the outpatient clinic. For these, the final revenue decreases significantly (*p* < 0.001) with each minute of treatment time in the outpatient clinic, which can be explained by the additional costs of treatment in the outpatient clinic. This is not true for outpatients, where the final revenue increases significantly (*p* < 0.001) with each minute of treatment in the outpatient clinic. To explain this, it must be said that additional services provided in the stationary per-case flat rate system are not additionally remunerated, however, in the outpatient TARMED tariff they are.

Our data shows that the mere presence of patients already causes a nursing effort for basic care, such as regular monitoring, conversations, assistance with personal hygiene, staff handovers, documentation, etc. Some of these tasks are omitted in the outpatient setting, are compensated for by relatives or are professionally covered by outpatient nursing services.

In addition, there are apportionment costs that are allocated proportionally to all cases, for example, the use of infrastructure or the provision of specialist staff at night. The nursing staff of the day clinic is responsible for the care and support of the partially stationary treated patients as well as for further laboratory and instrumental diagnostics. They also look after patients from other clinics, take care of short-term admissions (e.g., infusion therapies) and are the admission station for stationary treated patients, as the beds are often still occupied in the morning on the day of admission and surgery. Accordingly, the cost rate here is also significantly higher than that of the outpatient clinic, given increased upfront services and the corresponding infrastructure.

In the outpatient clinic, admission takes place directly next to the operating room, which is supervised by a small team specifically assigned for this purpose. The team does not have to take on any other tasks. After the preparation for surgery outside the operating area by the same team, the patient is handed over to the anesthesia team. After the operation, the patient is transferred to the recovery ward as usual. Thereafter, patients are discharged directly home after the recovery phase. There is no further change of the nursing team with transfer. The cost rate in the outpatient clinic is the lowest. Already Friedlander et al. described a significant cost advantage in the outpatient setting by avoiding the high costs of stationary care [20] in their publication from 2019, as did van den Oever et al. [35]. If it is also taken into account that the patients of the outpatient clinic are cared for the shortest in mean only 60 min by the nurses of the outpatient clinic with the lowest cost rate and in no other department, the operation time, and the total anesthesia time are also the lowest, the effects of process optimization are clearly shown. These optimized values of the treatment process explain the significantly increased revenue of 793.12 CHF in the outpatient clinic, due to the increased efficiency and the lower costs.

This disproportion is also reflected in the remuneration. The base payment for unilateral hernia care in the Swiss DRG system is 9650 CHF, which is multiplied by patient-specific factors to calculate the final payment [6]. The principle of DRG payment is that all costs must be covered by this lump sum. In contrast, the final fee for outpatient unilateral hernia care in the TARMED tariff is approximately 1912 CHF (with a slight variation depending on the surgical technique), whereby material costs can also be charged directly. Despite this large revenue advantage (9650 CHF vs. 1912 CHF) for stationary inguinal hernia care, it is overall loss-making due to higher costs [6]. These costs include, in addition to the supply costs, apportionment costs for administrative areas, which are added per case on a percentage basis (e.g., Requests for cost approval). In the Swiss healthcare system, there are legal requirements for documentation in the areas of SwissDRG`s and the TARMED tariff, among others, which lead to considerable administrative expenses that cannot be circumvented. Although stationary cases are loss-making, their higher share of pay-as-you-go costs reduces overall case costs more than profitable outpatient cases.

From a health economical and health policy perspective, a shift to less expensive treatment areas should be welcomed if it is done without compromising quality. The present study on inguinal hernias provides an example of the possibilities. When shifting to the outpatient sector, treatment costs can be reduced at case level. At system level, however, this presupposes that capacities in the inpatient sector are reduced while demand remains unchanged. This is the only way to effectively reduce costs in the overall system. Accordingly, there is a risk of selective optimization without any benefit for the system as a whole [36]. Moreover, this consolidation can only take place up to the minimum reserve capacities. For example, in an acute-somatic hospital, specialist staff must be available around the clock in the operating area, regardless of how often they are actually deployed. These overhead costs are then allocated as mentioned. The fewer cases that remain, the higher the percentage of apportioned costs. This can lead to a disbalance of the financial situation. Once this point has been reached, consolidation at the site level is necessary: in concrete terms, hospital sites would have to be reduced, for example.

With regard to the quality of treatment, a differentiated picture emerges: 9.09% of all outpatient planned procedures were unplanned stationary admissions and care due to postoperative complications (postoperative pain, intraoperative complications or complications affecting the cardiovascular system). This is 0.91% lower than described by Junaid et al. [19]. Outpatient readmission occurred within 30 days in 2.27% of previously hospitalized patients and in 4.79% of previously outpatient patients (stationary: 0%). In comparison with Junaid et al. [19], a deviation of −3.53% of the stationary treated patients is shown. Outpatients had a higher readmission rate of +2.29%. All readmissions were outpatient, no stationary retreatment was necessary. Nevertheless, it is advisable to reflect on this difference in the treatment team and, for example, to review patient selection and discharge management for optimization potential.

## 5. Limitations of the Study

A limitation of this study is the small number of cases overall and in the subgroups, which do not allow further subgroup analyses. Since emergency surgeries are usually performed in the stationary setting, there is an unintentional shift in the area of emergency treatment that has to be taken into account. However, the reimbursement for emergency surgery is also higher. Whether this increased reimbursement is sufficient to cover the additional costs must be examined in future studies. This study must also be viewed critically in terms of the unevenly distributed apportionment and emergency reserve capacities costs of the different departments. The outpatient clinic uses pre-existing infrastructures and draws on selective services of the overall system. For example, it uses the pre-existing nursing capacities of the recovery room. Even if the costs for this are taken into account, it would not be possible to isolate the process completely without additional costs. Moreover, a hospital’s pre-existing care services are part of its compensation. Accordingly, a comparison of organizational units with and without reserve capacities must be viewed critically. This is particularly the case because a shift from an organizational unit with reserve capacitiesto a process without reserve capacitieshas a negative impact on the profitability of the reserved processes, at least in the short term, until the structures there have been adapted again. In order to reduce health care costs, it therefore makes sense for as many treatments as possible to take place in an elective process without reserve capacities. As a result, organizational units with reserve capacitiesshould be concentrated as far as possible, on the one hand for quality assurance and on the other hand because they cannot be optimized at will. For example, by definition there must always be a 24 h service, regardless of how busy the specialist staff is. Finally, the allocation of overhead costs must also be mentioned critically: According to the accounting standard “REKOLE^®^” for hospitals in Switzerland, there is a certain amount of freedom to estimate overhead costs. These are not always complete and easy to allocate and are not always comparable between facilities. In addition, when billing, hospitals’ base rates may differ, making it difficult to generalize to all hospitals. Quality aspects also remain unconsidered. A complete assessment of all three objectives of the Health Insurance Act (KVG): effectiveness, appropriateness, and efficiency, cannot be derived. Another limitation is that this is a retrospective single center study. Prospective multicenter studies or meta-analyses could overcome these drawbacks and help to validate the findings. Quality of care parameters are only selectively assessed. A comprehensive picture of efficacy and appropriateness does not exist. For example, PROM (Patient Reported Outcome Measures) could make a complementary contribution and be considered in future studies.

## 6. Conclusions

Although inguinal hernia repair is a common operation in surgical practice, many hospitals find it difficult to provide cost-effective care. In our analysis we could show that cost effective elective unilateral hernia care in the outpatient clinic is possible with a profit of mean 793.12 CHF. As previously known, stationary unilateral hernia care is loss-making with a mean of −575.01 CHF. Decisive factors for cost efficiency are optimized processes in the operating room under compliance with predefined quality standards (anesthesia, surgical technique, operating time), as well as optimized and specialized nursing processes with minimal preoperative services and nursing time for the patient. The establishment of such a care structure should be in the interest of hospitals, insurance companies and the public governance under the current and the certainly increasing financial pressure in the future. At the same time, these optimizations pose a challenge to surgical training and structures with high levels of on-call services and pay-as-you-go costs.

The shift of stationary cases to the outpatient area has a greater impact on the overall system of a hospital. The effects must be evaluated on an ongoing basis. In particular, follow-up effects in other departments must be observed, such as whether stationary areas are not optimized in parallel. Only if it is ensured that the entire organization adapts to the new framework conditions is the development sensible in terms of health economics and business management. Otherwise, the savings will remain at the level of case costs, with a negative impact on the financial success of the hospital, since costs will merely be shifted in the face of lower revenues. In this regard, a more in-depth analysis at the hospital and care region level is appropriate, which evaluates the transformation to the outpatient sector.

The perspective of treatment quality shows a differentiated picture. On the one hand, the re-admissions of outpatients within 30 days should be analyzed in depth in order to improve the result. Quality transparency and public reporting are also to be further expanded to enable quality comparisons between hospitals. On the other hand, a continuation and an expansion of the quality evaluation is indicated, for example by means of PROMS. In the future, the focus on quality aspects should be integrated even more strongly into strategic considerations so that healthcare is not based solely on cost considerations.

## Figures and Tables

**Table 1:** Descriptive statistics of the whole sample and of the subgroups of hospital stay types.

*Variables*	*N*	*M*	*SD*	*Min*	*Q1*	*Md*	*Q3*	*Max*
	*All patients*							
Age	234	59.44	17.12	19	47	62	73	88
Anesthesia time	234	122.92	39.05	0	96	123	149	235
Surgery time	234	60.82	28.44	0	39	56	76	160
Stationary care time	234	165.30	223.66	0	0	0	361	929
Outpatient clinic care time	234	44.80	39.05	0	15	40	60	300
Recovery room care time	234	60.75	46.74	0	0	67	93	230
Doctors costs	234	855.87	822.12	72.47	320.65	521.05	1132.81	6280.61
Material costs	234	672.11	271.42	246.63	527.13	696.54	778.78	3089.02
Final Revenue	234	−95.36	1624.42	−4807.04	−851.07	33.21	586.36	16,207.03
	*Stationary treated patients*							
Age	88	65.41	16.38	20	53	71	77	88
Anesthesia time	88	146.64	34.08	61	122	143	169	235
Surgery time	88	76.05	31.38	30	50	73	97	160
Stationary care time	88	424.78	148.08	178	338	385	474	929
Outpatient clinic care time	40	47.25	34.40	15	15	45	68	150
Recovery room care time	76	98.97	36.26	36	72	96	119	230
Doctors costs	88	686.32	372.17	246.63	398.60	686.45	843.28	3089.02
Material costs	88	1595.77	926.86	456.66	1011.27	1407.37	1822.99	6280.61
Final Revenue	88	−575.01	2405.49	−4807.04	−1819.18	−869.06	159.22	16,207.03
	*Partially stationary treated patients*							
Age	107	55.55	16.01	20	43	59	66	88
Anesthesia time	106	124.43	25.70	65	105	124	142	198
Surgery time	106	57.80	21.77	21	41	56	72	120
Stationary care time	7	185.71	60.02	108	145	163	242	256
Outpatient clinic care time	102	61.40	35.92	10	40	48	91	300
Recovery room care time	98	68.31	27.31	1	51	68	85	190
Doctors costs	107	654.96	201.98	250.91	572.63	687.46	735.71	1398.09
Material costs	107	409.14	170.52	89.71	300.14	377.08	476.48	1102.31
Final Revenue	107	−24.73	690.76	−2042.53	−389.02	77.84	383.37	1804.31
	*Outpatient clinic patients*							
Age	39	56.62	18.29	19	43	62	72	79
Anesthesia time	39	68.44	10.57	48	60	70	75	88
Surgery time	39	36.23	10.72	19	28	37	43	68
Stationary care time	0	0.00	0.00	0	0	0	0	0
Outpatient clinic care time	39	59.77	30.50	25	40	60	60	135
Recovery room care time	0	0.00	0.00	0	0	0	0	0
Doctors costs	39	687.07	138.81	258.94	614.40	725.11	785.64	888.48
Material costs	39	411.99	253.72	72.47	248.06	292.41	516.24	1034.99
Final Revenue	39	793.12	493.90	−502.19	537.12	974.05	1161.35	1499.28

**Table 2:** Frequencies of categorical variables in the subgroups of stay types.

Variable	Categories	Stationary	Partially Stationary	Outpatient Center
Surgery type	Open surgery	37.5%	15.9%	2.6%
	Laparoscopic	62.5%	84.1%	97.4%
Incarcerated hernia	No	81.8%	100.0%	100.0%
	Yes	18.2%	0.0%	0.0%
Care in department	No	0.0%	93.5%	100.0%
	Yes	100.0%	6.5%	0.0%
Care in outpatient clinic	No	54.5%	4.7%	0.0%
	Yes	45.5%	95.3%	100.0%
Care in recovery room	No	13.6%	8.4%	100.0%
	Yes	86.4%	91.6%	0.0%
Insurance type	General	85.2%	92.5%	76.9%
	Private	14.8%	7.5%	23.1%

Notes. The percentages add up to 100% across the rows.

**Table 3:** Coefficients, *p* values, beta weights, and tolerances of the base model.

	*Estimate*	*SE*	*t Value*	*p Value*	β *Weight*	*Tolerances*
Age	−1.407	4.194	−0.335	0.738	−0.015	0.881
Doctors costs	0.960	0.156	6.162	0.000	0.486	0.493
Material costs	−0.359	0.303	−1.183	0.238	−0.060	0.768
Surgery time	−17.611	5.582	−3.155	0.002	−0.308	0.398
Anesthesia time	−10.443	4.705	−2.220	0.027	−0.251	0.344
Stationary care time	−0.706	0.697	−1.014	0.312	−0.097	0.406
Outpatient clinic care time	−6.083	1.882	−3.233	0.001	−0.146	0.861
Recovery room care time	4.210	1.790	2.352	0.020	0.121	0.756
Stay type (partially stationary)	1094.985	341.883	3.203	0.002	0.674	0.508
Stay type (outpatient clinic)	1200.142	435.829	2.754	0.006	0.739	0.508
Surgery type (laparoscopic)	329.625	226.627	1.454	0.147	0.203	0.674
Incarcerated hernia (yes)	−418.333	274.324	−1.525	0.129	−0.258	0.911
Insurance type (private)	−20.959	230.975	−0.091	0.928	−0.013	0.817

Notes. Multiple R-squared: 0.6697, adjusted R-squared: 0.6470; reference groups: stationary, open surgery, no incarceration, and general insurance.

**Table 4:** Coefficients, *p* values, and beta weights of the final model.

*Variable*	*Estimate*	*SE*	*t Value*	*p Value*	β *Weight*
(Intercept)	−2255.01	386.26	−5.84	0.000	−1.33
Doctors costs	2.62	0.42	6.25	0.000	1.32
Material costs	−0.23	0.63	−0.36	0.718	−0.04
Surgery time	−13.43	7.02	−1.91	0.058	−0.24
Anesthesia time	−17.40	4.92	−3.54	0.001	−0.42
Stationary care time	2.35	0.81	2.90	0.004	0.32
Outpatient clinic care time	−19.23	3.23	−5.95	0.000	−0.46
Recovery room care time	2.07	1.02	2.02	0.045	0.06
Age	24.73	6.40	3.87	0.000	0.26
Incarcerated hernia (yes)	3930.21	1752.31	2.24	0.027	2.42
Insurance (private)	−10,729.66	1182.83	−9.07	0.000	−6.61
Stay type (partially stationary)	2174.90	557.31	3.90	0.000	1.34
Stay type (outpatient clinic)	4654.06	2074.74	2.24	0.027	2.87
Surgery type (laparoscopic)	1516.01	421.52	3.60	0.000	0.93
Surgery type (laparoscopic): Stay type (partially stationary)	−2052.88	610.18	−3.36	0.001	−1.26
Surgery type (laparoscopic): Stay type (outpatient clinic)	−2486.49	973.02	−2.56	0.012	−1.53
Surgery time: Stay type (partially stationary)	26.35	14.99	1.76	0.081	0.46
Surgery time: Stay type (outpatient clinic)	112.04	55.18	2.03	0.044	1.96
Surgery time: Age: Stay type (partially stationary)	0.66	0.16	4.17	0.000	0.20
Surgery time: Age: Stay type (outpatient clinic)	0.54	0.57	0.96	0.339	0.16
Outpatient clinic care time: Stay type (partially stationary)	30.10	4.84	6.22	0.000	0.72
Outpatient clinic care time: Stay type (outpatient clinic)	37.05	6.80	5.44	0.000	0.89
Doctors costs: Stay type (partially stationary)	−3.42	0.51	−6.77	0.000	−1.73
Doctors costs: Stay type (outpatient clinic)	−5.16	3.05	−1.69	0.093	−2.61
Doctors costs: Surgery time: Stay type (partially stationary)	0.06	0.03	2.24	0.027	0.86
Doctors costs: Surgery time: Stay type (outpatient clinic)	0.10	0.06	1.63	0.106	1.37
Doctors costs: Anesthesia time: Stay type (partially stationary)	−0.07	0.02	−2.81	0.006	−1.30
Doctors costs: Anesthesia time: Stay type (outpatient clinic)	−0.08	0.07	−1.03	0.306	−1.50
Doctors costs: Insurance (private): Stay type (partially stationary)	−3.58	1.53	−2.35	0.020	−1.81
Doctors costs: Insurance (private): Stay type (outpatient clinic)	−2.49	1.00	−2.50	0.014	−1.26
Material costs: Stay type (partially stationary)	1.15	0.84	1.38	0.170	0.19
Material costs: Stay type (outpatient clinic)	−2.96	3.49	−0.85	0.398	−0.49
Material costs: Surgery time: Stay type (partially stationary)	0.12	0.02	5.53	0.000	0.56
Material costs: Surgery time: Stay type (outpatient clinic)	0.14	0.16	0.87	0.388	0.66
Material costs: Anesthesia time: Stay type (partially stationary)	−0.07	0.02	−3.53	0.001	−0.45
Material costs: Anesthesia time: Stay type (outpatient clinic)	−0.16	0.11	−1.50	0.137	−1.04
Material costs: Insurance (private): Stay type (partially stationary)	−27.85	5.09	−5.48	0.000	−4.65
Material costs: Insurance (private): Stay type (outpatient clinic)	−26.43	6.08	−4.35	0.000	−4.42
Age: Stay type (partially stationary)	−37.70	9.89	−3.81	0.000	−0.40
Age: Stay type (outpatient clinic)	−23.15	18.28	−1.27	0.208	−0.24
Insurance (private): Stay type (partially stationary)	9395.59	1537.24	6.11	0.000	5.78
Insurance (private): Stay type (outpatient clinic)	9205.35	1732.66	5.31	0.000	5.67
Insurance (private): Age: Stay type (partially stationary)	−308.22	73.39	−4.20	0.000	−3.25
Insurance (private): Age: Stay type (outpatient clinic)	−204.63	58.74	−3.48	0.001	−2.16

Notes. Multiple R-squared: 0.9782, adjusted R-squared: 0.9605; reference groups: stationary, open surgery, no incarceration, and general insurance.

**Table 5:** Impact of outpatient clinic care time in interaction with stay type.

*Stay Type*	*Variable*	*Estimate*	*SE*	*t Value*	*p Value*	β *Weight*
Stationary	Outpatient clinic care time	−19.76	3.58	−5.51	0.000	−0.48
Partially stationary	Outpatient clinic care time	10.34	1.83	5.65	0.000	0.25
Outpatient clinic	Outpatient clinic care time	17.29	4.13	4.19	0.000	0.42

Note. Reported are the simple slopes of the interaction of outpatient clinic care time with stay type.

## Data Availability

The datasets used and/or analyzed during the current study are available from the corresponding author on reasonable request.

## Abbreviations

PROMS	Patient Reported Outcome Measure
EHS	European Hernia Society
IEHS	International Endohernia Society
GZO	Gesundheitszentrum Zürich Oberland
CHF	Swiss Franc
SwissDRG	Swiss Diagnosis Related Groups
ASA	American Society of Anesthesiologists
DRG	Diagnosis Related Groups
TARMED	*(Swiss Federal Health Insurance Act)*
PWC	PricewaterhouseCoopers
BFS	Bundesamt für Statistik *(Federal office for statistics)*
GDP	Gross Domestic Product
EUR	Euro
WHO	World Health Organization
OR	Operating Room
